# New-Onset Psoriasis Induced by Durvalumab

**DOI:** 10.7759/cureus.48453

**Published:** 2023-11-07

**Authors:** Rajaa Shoukfeh, Reem Yassine, Dilara Turk, Meena Moossavi, April Nofzinger

**Affiliations:** 1 Department of Medicine, Wayne State University, Detroit, USA; 2 Department of Osteopathic Medicine, Michigan State University, East Lansing, USA; 3 Department of Dermatology, Wayne State University, Detroit, USA; 4 Department of Hematology, Veterans Affairs Medical Center, Detroit, USA

**Keywords:** durvalumab, medication side-effects, onco-immunology, de novo psoriasis, drug-induced psoriasis, dermatology case report, clinical dermatology

## Abstract

Durvalumab is an immune checkpoint inhibitor (ICI) belonging to the anti-programmed death-ligand 1 (PD-L1) class, and it is used in the treatment of various end-stage malignancies. Immune checkpoint inhibitors are associated with various systemic and cutaneous adverse events. Psoriasiform drug eruptions have been clinically observed in patients who have a personal history of psoriasis being treated with ICIs. We present a unique case of de novo psoriasis in a patient being treated for poorly differentiated adenocarcinoma of the lung. The patient responded well to topical treatment and did not require discontinuation of durvalumab. Our case highlights the importance of clinician familiarity with psoriasis presentation, its association with durvalumab therapy, and treatment options for durvalumab-induced psoriasis.

## Introduction

Immune checkpoint inhibitors (ICIs) are novel agents used for the treatment of various malignancies. These drugs include anti-programmed death 1 (PD-1) and anti-programmed death-ligand 1 (PD-L1), which inhibit the programmed cell death protein 1 and the programmed cell death ligand, respectively [1]. The PD-1/PD-L1 pathway is involved in the control of inflammation and functions to maintain an immunosuppressive environment; hence, anti-PD1/PD-L1 therapy enhances the anti-tumor response [1]. Durvalumab is an anti-PD-L1 agent used in the treatment of advanced or metastatic urothelial carcinoma, extensive stage small cell lung cancer, and unresectable stage III non-small cell lung cancer in adults who did not have disease progression following radiation and chemotherapy [2]. Though they offer potent therapeutic benefits, ICIs also entail a wide range of immune-related adverse events owing to their activation of cytotoxic T cells. A few systemic adverse events associated with ICIs are hepatitis, thyroiditis, pneumonitis, colitis, or pancreatitis [1]. Dermatologic toxicities are the most prevalent adverse event, presenting as lichenoid dermatitis, vitiligo, and psoriasis [1]. In a randomized clinical trial, it was shown that 12.2% of patients undergoing durvalumab treatment developed a rash, compared to 7% in the placebo group [2]. Patients who develop psoriasis following ICIs have a history of psoriasis; however, there has been one reported case of durvalumab-induced de novo psoriasis [3]. Both personal and related family histories of psoriasis are important risk factors to be considered before initiating treatment [4]. In this article, we report an unusual presentation of de novo durvalumab-induced psoriasis.

## Case presentation

**Figure 1:**
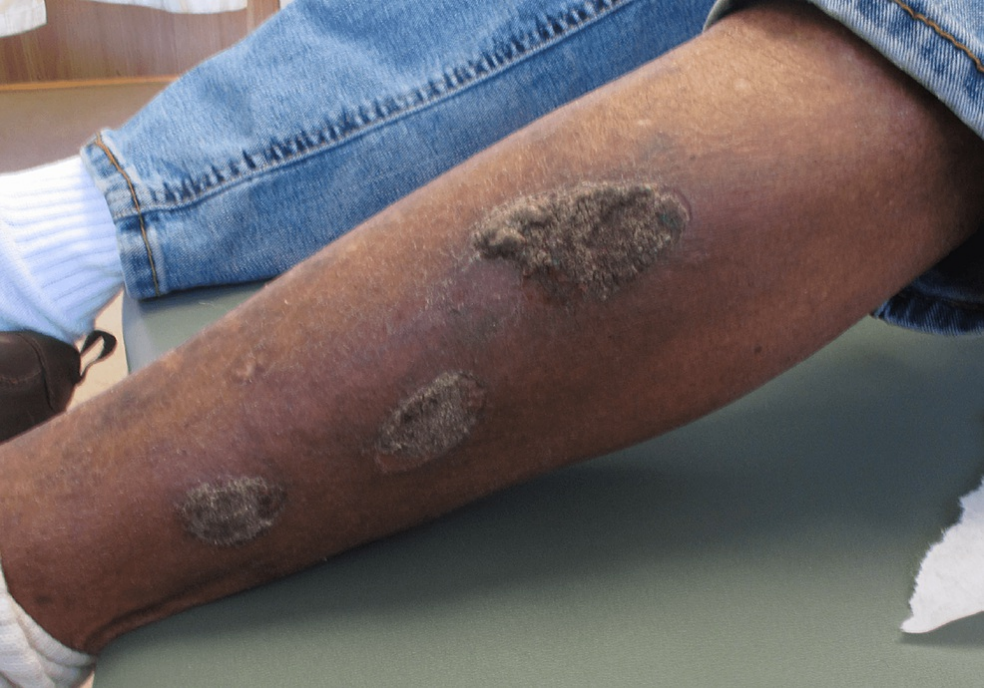
Clinical presentation of psoriasis induced by durvalumab; left lateral leg with three well-demarcated brown hyperkeratotic plaques are seen.

A 74-year-old man with stage III poorly differentiated adenocarcinoma of the lung being treated with durvalumab after chemotherapy and radiation presented to our dermatology clinic. The patient had a personal history of atopic dermatitis during childhood; however, he denied having any personal or family history of psoriasis or any other autoimmune or rheumatologic disease. He had no significant comorbidities. A medication review showed no psoriasis-inducing drugs. After eight months of treatment with durvalumab, he developed multiple enlarging plaques on his left lower extremity and back. The patient described the lesions as mildly itchy and stated that the lesions were slowly enlarging. Clinical examination revealed three brown hyperkeratotic plaques on his left lateral lower leg and one plaque on the mid-lower back (Figure 1).

**Figure 2:**
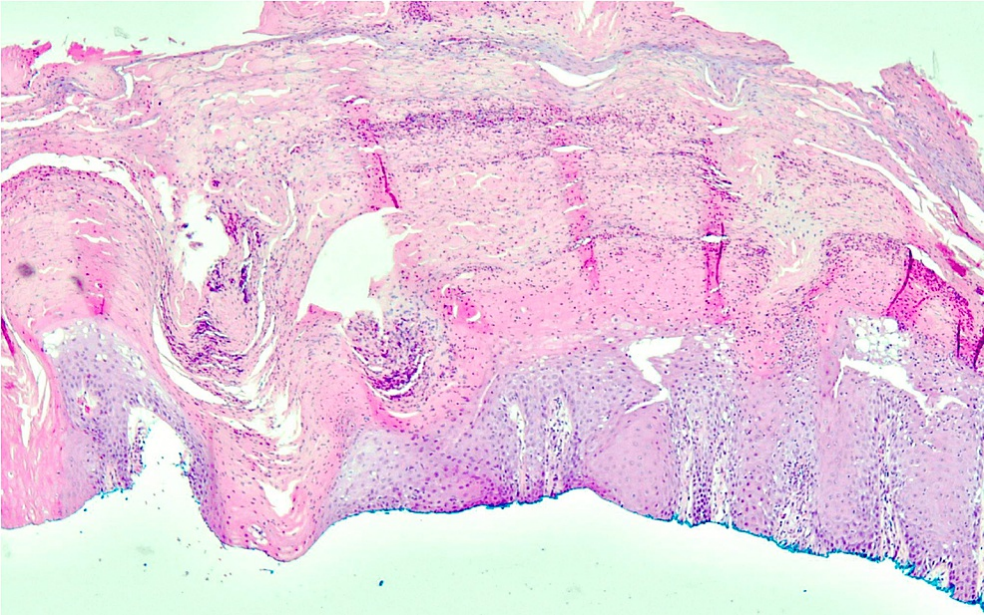
Histological presentation of psoriasis induced by durvalumab: psoriasiform acanthosis with parakeratosis and presence of Munro microabscesses (hematoxylin-eosin, 10X magnification)

Given their unusual appearance and size, a shave biopsy of the largest lesion on the patient’s left lower leg was performed to confirm the diagnosis. The skin biopsy of the lesion revealed psoriasiform acanthosis with parakeratosis and the presence of Munro microabscesses, which was consistent with psoriasis (Figure 2).

Clinically, the patient’s presentation was consistent with rupioid psoriasis. Given that the patient’s psoriasis presented after the initiation of durvalumab, it is presumed that the patient developed psoriasis induced by durvalumab. Another possible differential diagnosis is seborrheic keratosis, which presents with waxy or scaly stuck-on plaque. This clinical presentation is similar to that of our patient; however, histopathology of seborrheic keratosis would reveal keratin-filled invaginations and small horn cysts. The patient’s psoriasis was limited to only a few lesions on the body, therefore, he was treated with clobetasol ointment to apply to the plaques twice a day. The patient’s oncologist continued his durvalumab for another four months, completing one year of therapy for the treatment of his lung adenocarcinoma. At the patient’s follow-up after durvalumab completion, his lesions had decreased in size, and he did not report any new plaques.

## Discussion

Durvalumab is currently used for the treatment of a variety of malignancies, including metastatic non-small cell lung cancer, locally advanced or metastatic urothelial carcinoma, extensive-stage small-cell lung cancer, and metastatic Merkel cell carcinoma [5,6]. The recurrence or new onset of psoriasis with ICI treatment may be related to the impact of PD-1 inhibitors on the Th1/Th17 pathways, which results in an over-expression of interleukin (IL)-17 and the inflammatory cascade [5].

Existing literature supports the need for strict skin surveillance for patients on ICIs, such as durvalumab, who have a personal or family history of psoriasis [7]. However, our case brings to light the possibility of de novo psoriasis onset occurring with durvalumab therapy.

This case is unique in that previously reported ICIs-induced drug eruptions often present as guttate or plaque psoriasis, whereas rupioid psoriasis, characterized by a hyperkeratotic cone-shaped plaque, has not been reported. While our patient did not have a personal history of psoriasis, he did report having a different inflammatory skin condition, atopic dermatitis, in childhood. Current literature supports the idea that patients with a history of autoimmune or inflammatory disease are significantly susceptible to immune-mediated adverse events when treated with ICIs [6].

Psoriasis flares occurring during ICI therapy typically do not warrant immunotherapy discontinuation due to the severity of the underlying malignant conditions being treated with ICIs [7, 8]. Current treatment recommendations for psoriasis flares during immunotherapy include either topical and systemic older drugs or new biologic treatment options [7]. The National Comprehensive Cancer Network (NCCN) 2021 guidelines recommend routine examination of the skin and mucosa for patients treated with ICIs who have a medical history of immune-related skin disorders [9]. For mild maculopapular skin eruptions, the NCCN recommends the use of an oral antihistamine, topical emollient, and moderate-potency topical corticosteroid with continuation of ICI therapy [9]. Our case highlights the importance of clinician familiarity with psoriasis induced by durvalumab and the need for continued clinical awareness of cutaneous adverse reactions to ICIs.

## Conclusions

Immune checkpoint inhibitors have shown remarkable efficacy in treating various cancers, but they can also induce rare side effects, such as exacerbating or even causing psoriasis, in patients with no prior history of this skin condition. When confronted with psoriasis flares during ICI therapy, healthcare providers often recommend a combination of treatment approaches. In mild cases, management can involve the use of oral antihistamines to alleviate itching, topical emollients to maintain skin hydration, and mild-potency topical corticosteroids to reduce inflammation. Importantly, the continuation of ICI therapy is typically advised to ensure that the patient receives the full benefit of cancer treatment while managing psoriasis symptoms. In more severe cases, biologics, immunosuppressive medications, or phototherapy may be necessary to control the psoriasis flare, always with a careful balance between treating the skin condition and maintaining the efficacy of the immune checkpoint inhibitors in fighting cancer.
